# Supplementary material for: The Influence of Kerosene on Microbiomes of Diverse Soils
Source: Life (Basel). 2022 Jan 31;12(2):221. doi: 10.3390/life12020221 (PMC8878009; doi:10.3390/life12020221)
Supplement: Supplementary file 1 [file life-12-00221-s001.zip › life-1537659-supplementary.pdf]

Article

The Influence of Kerosene on Microbiomes of Diverse Soils

Pavel V. Shelyakin [1,2,†], Ivan N. Semenkov [3,†,*], Maria N. Tutukina [1,4,5,†], Daria D. Nikolaeva [1,4,†], Anna V. Sharapova [3], Yulia V. Sarana [4], Sergey A. Lednev [3], Alexander D. Smolenkov [6], Mikhail S. Gelfand [1,4,‡], Pavel P. Krechetov [3,‡] and Tatiana V. Koroleva [3,‡]

[1] Institute for Information Transmission Problems (Kharkevich Institute), Russian Academy of Sciences, 127051 Moscow, Russia; f.serval@gmail.com (P.V.S.); m.tutukina@skoltech.ru (M.N.T.); mikhail.gelfand@gmail.com (M.S.G.)
[2] Department of Computational Biology, N.I. Vavilov Institute of General Genetics, Russian Academy of Sciences, 119333 Moscow, Russia; f.serval@gmail.com (P.V.S.)
[3] Faculty of Geography, M.V. Lomonosov Moscow State University, 119991 Moscow, Russia; avsharapova@mail.ru (A.V.S.); sled1988@mail.ru (S.A.L.); krechetov@mail.ru (P.P.K.); korolevat@mail.ru (T.V.K.)
[4] Center of Molecular and Cellular Biology, Skolkovo Institute of Science and Technology, 121205 Moscow, Russia; daria.nikolaeva@skoltech.ru (D.D.N.); Yuliya.Sarana@skoltech.ru (Y.V.S.)
[5] Lab of Functional genomics and cellular stress, Institute of Cell Biophysics RAS, 142290, Pushchino, Moscow region, Russia; m.tutukina@skoltech.ru (M.N.T.)
[6] Faculty of Chemistry, M.V. Lomonosov Moscow State University, 119991 Moscow, Russia; smolenkov@bk.ru
* Correspondence: semenkov@geogr.msu.ru
† These authors share the first authorship.
‡ These authors share the last authorship.

Supplementary Table S1. Meteorological conditions for the period of the field experiment with Albic Luvisols and Fibric Histosols.

Year	Month	Air temperature,°C	Air humidity,°C	Soil surface temperature, °C	Precipitation, mm
2020	June	18.7	72	n.a.	368
	July	17.9	77	n.a.	210
	August	16.7	75	n.a.	83
	September	13.0	72	4.2	128
	October	9.1	73	2.4	69
	November	1.6	87	n.a.	98
	December	-4.5	87	n.a.	64
2021	January	-6.2	89	n.a.	132
	February	-11.0	82	n.a.	145
	March	-2.2	74	n.a.	62
	April	6.7	66	2	76
	May	13.2	66	6.4	198
	June	19.0	70	14.4	127

Data are processed from the meteorological station Maly Yaroslavets (https://rp5.ru/Weather_in_Maloyaroslavets), which is closest to the study area.

Supplementary Table S2. Methods of soil chemical analyses used.

Parameters	Methods and equipment	Reference
Kerosene	Extraction with anhydrous sodium sulfate and methylene chloride for 15 min in an ultrasonic bath. Filtration. Gas chromatography: theAgilent 7890 V gas chromatograph by Agilent Technologies (Santa Clara, California, the USA) equipped with the 5977 A quadrupole mass-spectrometric detector	[1]
Total organic carbon	Wet dichromate oxidation and spectrocolorimetery	[2]
Loss on ignition	Loss on ignition at 525°C	[2]
pH	Soil: water ratio 1:5. Potentiometry: pH-meter 'I-160MI' (Izmeritelnaya technika, Moscow, Russia)	[2]
NO_3^-	Soil: water ratio 1:5. Photometry (salicylic acid; wavelength 410 nm)	[2]
NH_4^+	Extraction with 1 mol L-1 KCl. Photometry (indophenol; wavelength 655 nm)	[2]
Pav	Extraction with 0.2 M HCl (Histosols and Luvisols) and 0.2 M $(NH_4)_2CO_3$ (soil: solution ratio 1:5 and 1:50 for A-horizons and O-horizons, respectively), spectrophotometry (wavelength 710 nm)	[2–4]
Kav	Extraction with 0.2 M HCl (Histosols and Luvisols) and 0.2 M $(NH_4)_2CO_3$ (soil: solution ratio 1:5 and 1:50 for A-horizons and O-horizons, respectively), spectrophotometry. The extracted potassium was measured by the inductively coupled plasma atomic emission spectrometry. Agilent 720 ICP-OES (Agilent Technologies, Malaysia).	[2–4]
CEC	Magnesium acetate method with complexometric determination of Mg with EDTA.<br>1. Saturation:<br>Saturation with 0.25 mol L-1 magnesium acetate (pH 7.0); agitation 30 min; decantation 5 min; filtration;<br>Saturation with 0.5 mol L-1 magnesium acetate (pH 7.0); agitation 15 min; decantation 5 min; filtration;<br>Saturation with 0.25 mol L-1 magnesium acetate (pH 7.0); shaking; filtration;<br>2. Twice washing-out by distilled water<br>3. Displacement: 0.5 mol L-1 KC1.	[2,5]
Moisture	Gravimetry after drying at a temperature of 105°C	[2]

1. Bolotnik, T.A.; Timchenko, Y. V.; Plyushchenko, I. V.; Levkina, V. V.; Pirogov, A. V.; Smolenkov, A.D.; Popik, M. V.; Shpigun, O.A. Use of Chemometric Methods of Data Analysis for the Identification and Typification of Petroleum and Petroleum Products. *J. Anal. Chem.* **2019**, *74*, 1336–1340, doi:10.1134/S1061934819130045.
2. Pansu, M.; Gautheyrou, J. *Handbook of Soil Analysis*; 2006;
3. SS 54650-2011 Soils. Determination of mobile phosphorus and potassium compounds by Kirsanov method modified by CINAO 2011, 11.
4. SS-26205-91 Soils. Determination of mobile compounds of phosphorus and potassium by Machigin method modified by CINAO 1991, 10.
5. SS-17.4.4.01-84 Nature protection. Soils. Methods for determining the capacity of cation exchange 1984.

Supplementary Table S3. Temporal changes in concentration of kerosine during the pot (P) and field (F) experiment (g/kg).

Kerosene load, g/kg	Experiment	Days after kerosene treatment			
		**3**	**90**	**180**	**360**
		**Albic Luvisols**			
0	P	<0.1	<0.1	<0.1	<0.1
	F	<0.1	<0.1	<0.1	<0.1
1	P	0.33±0.06	<0.1	<0.1	<0.1
	F	<0.1	<0.1	<0.1	<0.1
5	P	3.7±0.6	**0.19±0.03**	<0.1	<0.1
	F	1.1±0.3	<0.1	<0.1	<0.1
10	P	6.0±0.7	**2.5±0.3**	**0.27±0.01**	<0.1
	F	8.3±0.6	**0.26±0.05**	<0.1	<0.1
25	P	21.1±1.7	15.3±4.4	**11.0±1.5**	**0.27±0.05**
	F	8.9±1.4	**0.80±0.18**	**0.12±0.02**	**0.12±0.02**
100	P	78.3±0.9	**85.5±6.4**	77.9±1.6	**1.1±0.3**
	F	26.5±5.7	**2.2±0.1**	**0.37±0.10**	**0.32±0.01**
		**Dystric Arenosols**			
0	P	<0.1	<0.1	<0.1	<0.1
1	P	0.37±0.09	<0.1	<0.1	<0.1
5	P	4.3±0.4	**0.28±**	<0.1	<0.1
10	P	6.5±0.5	**2.8±**	**0.27±0.03**	<0.1
25	P	23.9±0.6	**17.8±**	**13.5±0.8**	**0.27±0.05**
100	P	80.2±6.9	65.8±	77.9±2.7	**2.3±0.4**
		**Fibric Histosols**			
0	F	<0.1	<0.1	<0.1	<0.1
1	F	0.11±0.01	<0.1	<0.1	<0.1
5	F	1.9±0.7	<0.1	<0.1	<0.1
10	F	7.1±1.4	**0.22±0.05**	<0.1	<0.1
25	F	7.8±0.9	**0.87±0.20**	<0.1	<0.1
100	F	8.3±1.2	**1.2±0.3**	**0.20±0.13**	**0.12±0.03**

Significant ($p<0.05$) differences with the initial variant are in bold.

Supplementary Table S4 Temporal changes in soil physicochemical properties during the pot (P) and field (F) experiment.

Kerosene load, g/kg	Experiment	pH				TOC,%				Moisture, %				CEC, mg(+)/100g				Pav, mg/kg				NO3-, mg/kg				NH4+, mg/kg				Kav, mg/kg			
		3	90	180	360	3	90	180	360	3	90	180	360	3	90	180	360	3	90	180	360	3	90	180	360	3	90	180	360	3	90	180	360
**Albic Luvisols**																																	
0	P	7.2	7.3	6.3	5.8	3.9	3.3	3.6	3.5	18	19	19	21	18	18	13	41	62	65	70	118	25	0.9	7	15	71	83	11	16	148	202	148	265
	F	6.4	6.3	5.8	6.3	4.5	6.1	0.1	8.0	34	29	26	47	25	15	14	12	54	105	46	85	12	22	60	<5	8	45	3	2	144	141	135	18
1	P	6.3	6.8	6.1	5.5	4.4	3.3	2.8	3.5	18	19	19	16	18	18	11	39	67	65	81	115	23	0.6	13	50	86	103	42	65	146	203	133	260
	F	5.7	5.9	5.4	6.2	3.6	2.5	1.4	7.0	34	26	25	55	26	18	19	15	93	116	36	177	20	3.7	53	107	19	42	13	4	112	150	161	20
5	P	5.6	6.9	6.6	6.0	3.2	4.1	2.8	5.1	18	19	19	7	18	18	15	30	87	65	129	116	20	0.6	0.5	<0.05	100	127	131	67	166	224	188	291
	F	6.2	5.6	6.4	6.4	5.7	5.5	3.4	8.3	28	31	30	46	28	21	19	12	111	148	53	297	2.2	24	28	10	127	10	45	5	155	235	210	26
10	P	5.5	6.3	6.4	6.0	4.0	5.9	3.6	4.8	18	19	19	17	18	18	13	42	89	67	111	128	25	2.7	0.7	<0.05	0	0	113	70	163	227	135	282
	F	6.0	6.2	6.8	6.4	6.6	4.3	4.9	7.8	32	29	30	47	34	26	15	12	130	121	66	70	1.6	1.8	23	<5	179	57	44	4	187	231	257	16
25	P	5.5	6.3	6.1	5.5	5.7	6.1	3.5	5.0	19	20	19	17	19	19	13	24	82	69	131	146	19	2.3	0.8	<0.05	100	131	105	75	155	177	137	275
	F	5.9	6.3	6.3	6.4	5.7	8.5	3.6	7.0	26	34	30	48	26	22	21	12	153	108	72	62	1.5	2.2	76	<5	157	39	51	20	187	242	264	57
100	P	5.9	6.8	6.0	4.9	11.1	9.7	8.5	5.8	23	24	24	17	23	21	13	37	67	66	109	141	30	0.5	0.9	<0.05	77	90	101	63	140	163	333	269
	F	5.8	6.6	7.1	6.6	5.2	6.4	10.0	8.5	34	35	34	62	16	22	22	13	112	93	70	131	1.5	1.7	3.7	<5	124	6	45	3	190	248	263	40
**Dystric Arenosols**																																	
0	P	8.6	9.6	8.9	9.3	0.40	0.55	0.12	0.36	4.3	4.7	4.5	3.5	8	7	7	28	14	25	12	83	31	0.5	1.7	<0.05	4	3.2	8.2	6.0	95	119	221	121
1	P	8.5	9.5	9.2	9.3	0.58	0.31	0.04	0.23	4.1	3.8	4.0	2.6	9	7	9	30	13	26	11	65	30	2.7	2.4	<0.05	5	5.4	8.5	3.3	99	125	225	126
5	P	8.5	9.5	8.9	9.2	0.08	0.37	0.74	0.36	4.5	5.2	4.9	2.1	7	8	8	15	16	30	12	70	0.3	0.3	0.2	<0.05	0	5.8	11.4	3.0	97	113	238	125
10	P	8.6	9.6	9.0	9.1	0.59	1.08	0.04	0.43	4.6	5.5	5.1	0.1	7	8	8	13	15	29	8	86	<0.05	0.3	0.3	<0.05	89	5.3	8.2	3.2	99	110	271	121
25	P	8.5	9.4	8.8	9.0	0.79	1.10	0.70	0.64	5.3	5.9	5.7	1.3	10	9	9	34	16	28	20	87	13	0.5	0.6	<0.05	7	4.7	7.1	2.6	97	117	229	115
100	P	8.4	9.4	8.7	9.1	2.24	3.20	2.51	0.33	10.3	10.0	10.3	1.9	8	8	6	14	17	30	14	76	25	0.5	0.5	<0.05	5	5.3	8.1	7.9	91	119	215	124
**Fibric Histosols**																																	
0	F	4.1	4.7	5.0	5.0	11	98	71	98	509	91	89	950	19	45	17	11	94	70	47	227	1.7	2	2.7	<5	133	51	75	36	87	83	136	45
1	F	4.4	4.7	5.0	4.8	12	96	90	98	234	85	88	1150	38	13	14	9	59	82	47	203	2	1.9	6	<5	80	40	60	28	80	149	70	49
5	F	4.5	4.8	4.9	4.8	7	98	92	98	255	89	87	1060	17	28	12	11	53	59	39	166	1.8	1.7	3.1	<5	39	44	37	34	118	75	85	40
10	F	4.0	5.1	4.8	5.3	9	98	58	97	236	86	89	1080	43	30	11	9	69	67	53	110	2.2	1.9	2.6	<5	99	73	63	28	80	102	74	15
25	F	4.2	4.4	4.9	6.0	8	99	53	96	200	90	90	850	44	17	13	10	53	30	31	83	1.7	1.8	4.6	<5	41	36	38	23	79	50	58	12
100	F	4.3	4.8	4.7	6.1	9	98	80	97	258	91	88	1300	22	13	27	10	34	22	25	105	1.7	2	8	<5	60	35	29	17	71	58	66	9

Supplementary Table S5. Significance of differences between the topsoil samples (16S rRNA) in a pot (P) and field (F) experiments within specified groups. Assessed using PERMANOVA on weighted UNIFRAC beta-diversity.

**Kerosene load**	***p*-value (pass fdr correction: +/-)**							
	Albic Luvisols (P)		Albic Luvisols (F)		Dystric Arenosols (P)		Fibric Histosols (F)	
	V3V4	V4V5	V3V4	V4V5	V3V4	V4V5	V3V4	V4V5
0	**0.0001+**	**0.0003+**	0.4364-	0.2768-	**0.0001+**	**0.0001+**	*0.2095-	*0.3722-
1	**0.0001+**	**0.0002+**	0.9196-	0.8740-	**0.0002+**	**0.0001+**	*0.4111-	*0.4778-
5	**0.0002+**	**0.0001+**	**0.0089+**	*0.0508-	**0.0001+**	**0.0001+**	*0.6556-	*0.8302-
10	**0.0002+**	**0.0001+**	*0.0619-	0.0496-	**0.0002+**	**0.0002+**	*0.6333-	*0.6698-
25	**0.0001+**	**0.0001+**	**0.0119+**	*0.0159-	**0.0001+**	**0.0001+**	*0.1095-	*0.5016-
100	**0.0059+**	**0.0038+**	***0.0111+**	***0.0063+**	**0.0001+**	**0.0001+**	*0.7889-	*0.3333-
**Day**								
3	**0.0001+**	0.1296-	0.0371-	0.0852-	**0.0018+**	**0.002+**	0.2797-	0.3602-
90	**0.0001+**	**0.0001+**	**0.0001+**	0.0348-	**0.0001+**	**0.0001+**	0.0972-	0.1065-
180	**0.0001+**	**0.0001+**	**0.0172+**	0.0249-	**0.0001+**	**0.0001+**	0.1592-	0.0195-
360	**0.0001+**	**0.0001+**	**0.0021+**	**0.0023+**	**0.0001+**	**0.0001+**	*0.2903-	*0.5083-

* Due to insufficient number of samples in some groups, less than 9999 permutations were done during PERMANOVA, which limits the precision of p-values.

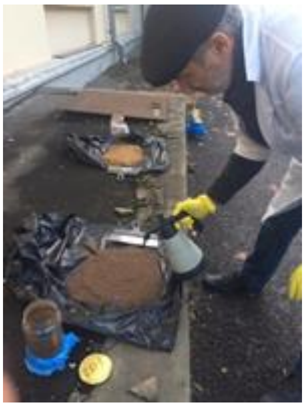

A

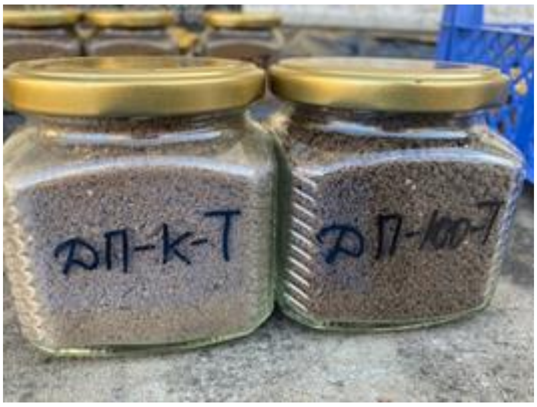

B

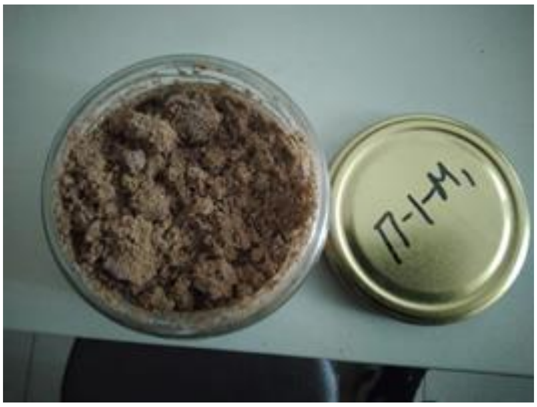

C

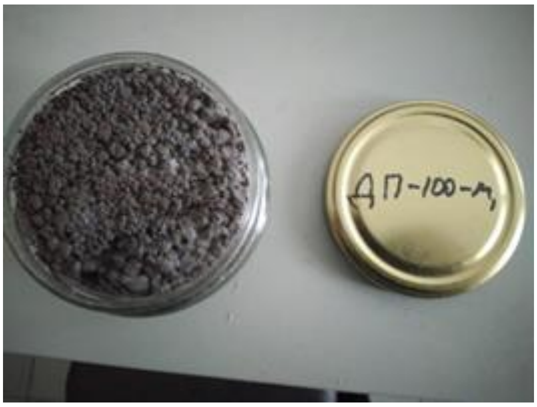

D

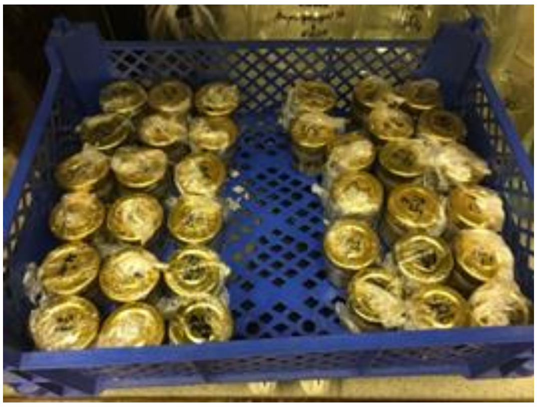

E

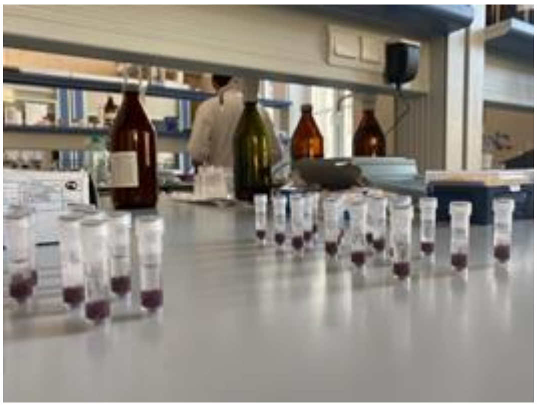

F

Supplementary Figure S1. Soil treatment with kerosene and soil sampling. A, Treatment with kerosene of soil stored in a plastic bag (11 g/kg load). B, subsamples placed in 400 cm3 glass containers with hermetically sealed iron lids. C, Structure of the A-horizon of Arenosols preserved after kerosene treatment. D, Structure of the A-horizon of Luvisols preserved after kerosene treatment. E, Soil subsamples ready for transportation to the laboratory. F, Soil subsamples for the DNA isolation

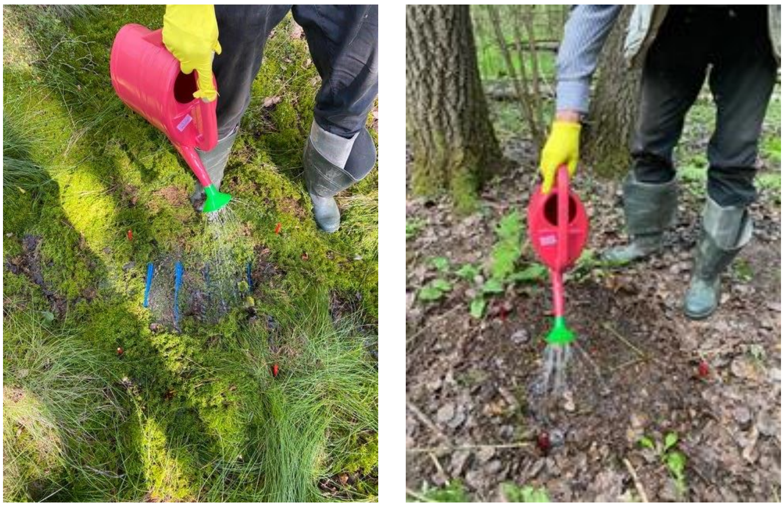

Fibric Histosols Albic Luvisols

Supplementary Figure S2. Experimental plots of 50×50 cm in size.

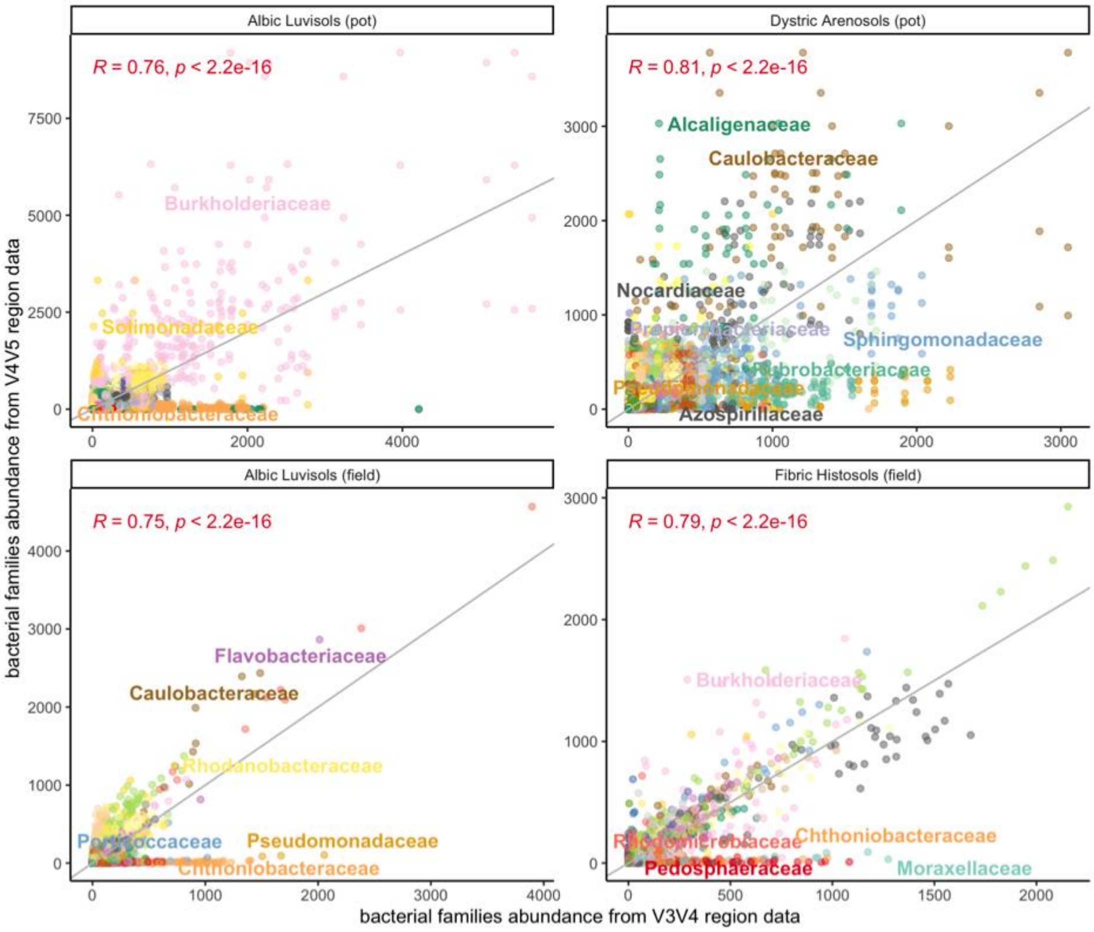

Supplementary Figure S3. Bacterial abundances estimated using different 16S rRNA.

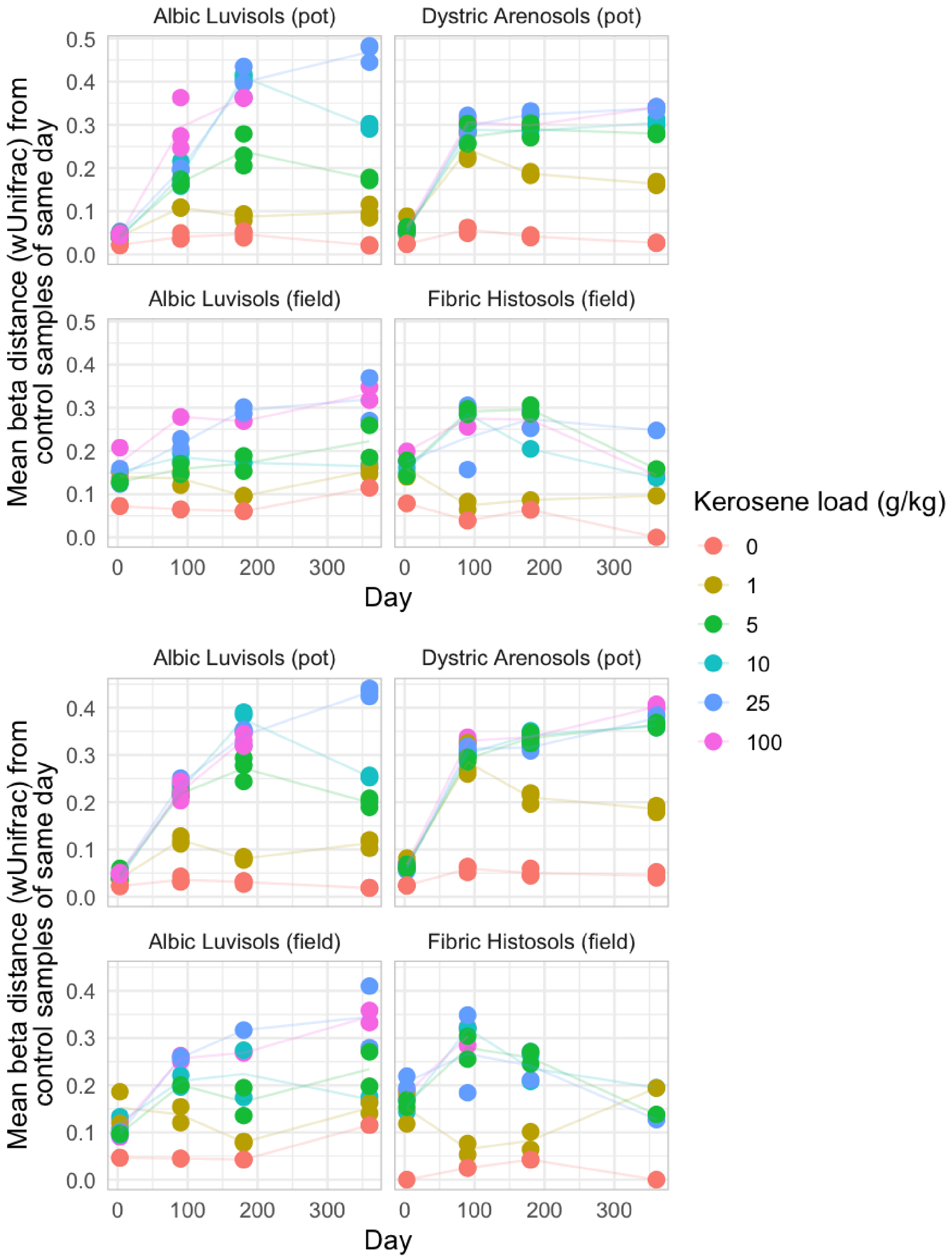

Supplementary Figure S4. Mean beta distance (weighted UNIFRAC) of microbiomes to the microbiomes from the control samples at the same day.

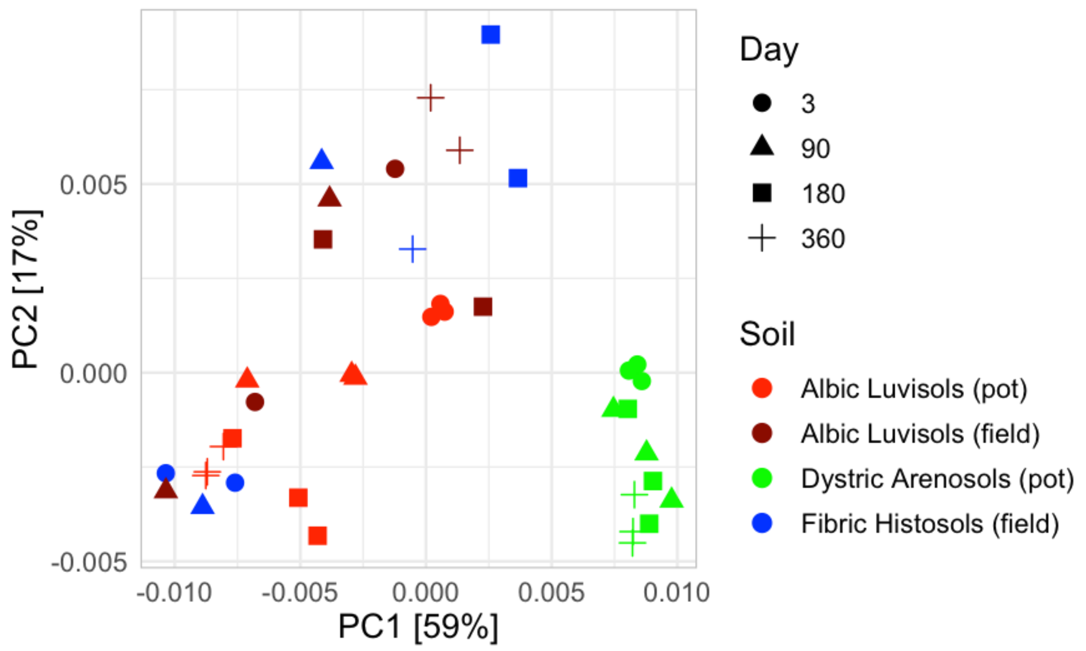

Supplementary Figure S5. PCA plot on fractions of abundance of MetaCyc pathways in samples without added kerosene, based on Picrust2 predictions for the V3V4 region.

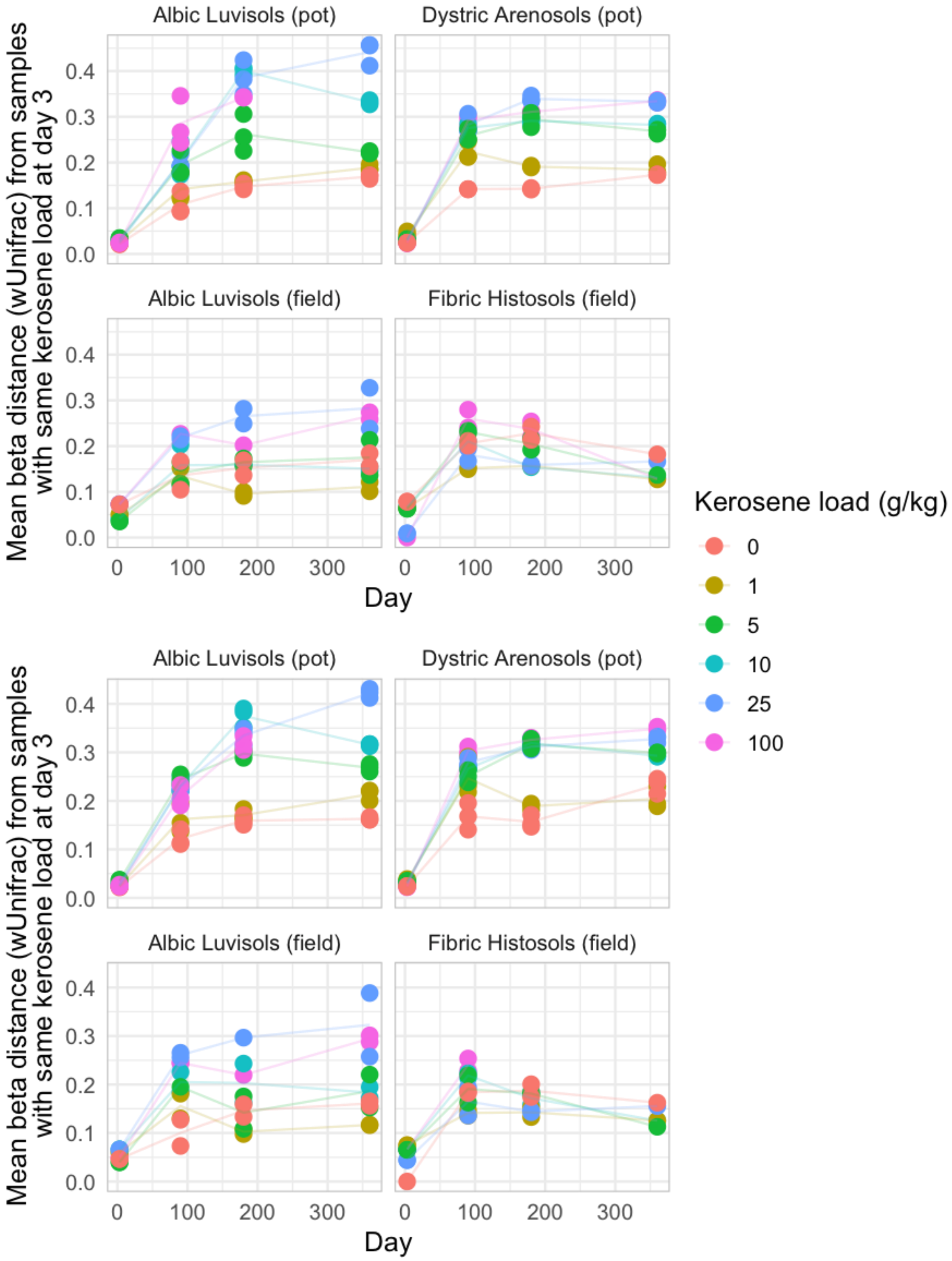

Supplementary Figure S6. Mean beta distance (weighted UNIFRAC) of microbiomes to the microbiomes from the samples with the same kerosene load at day 3.

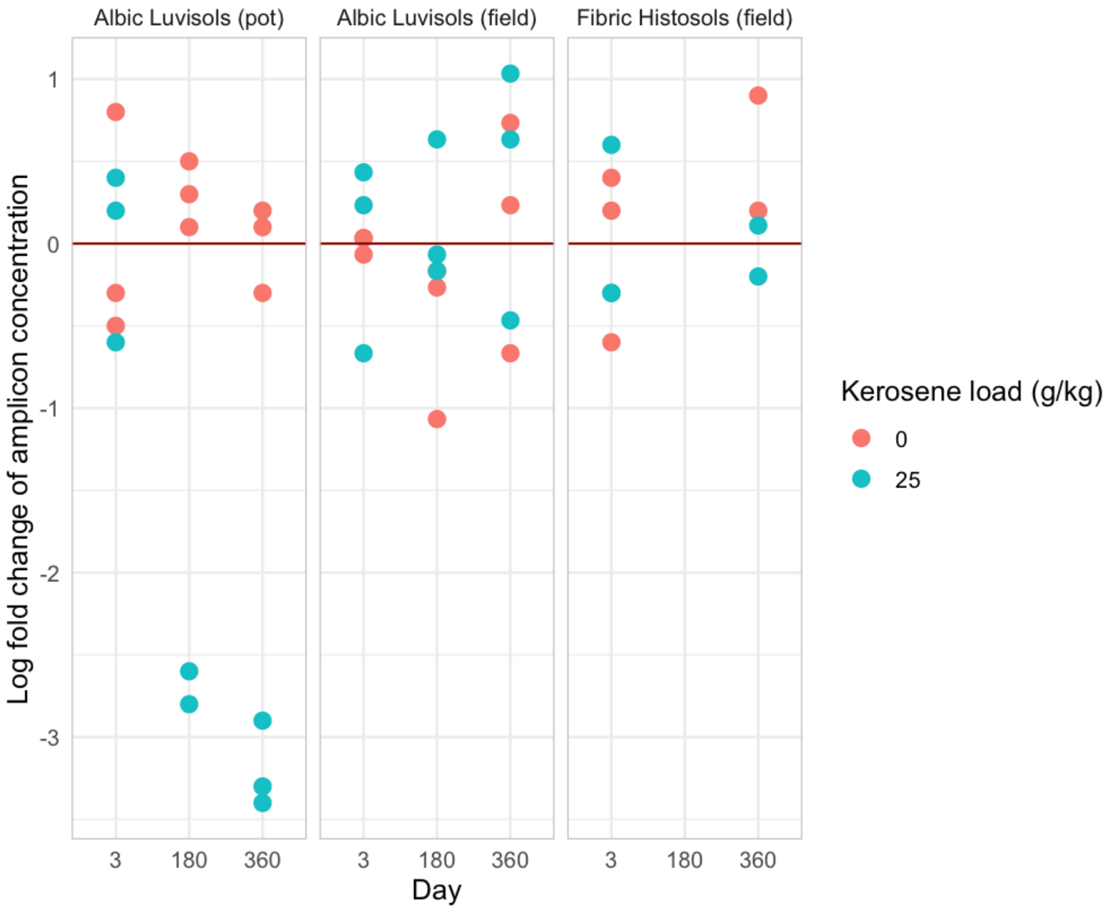

Supplementary Figure S7. Log fold change of the amplicon concentration estimated with qPCR relative to the mean amplicon concentration in control samples at day 3.

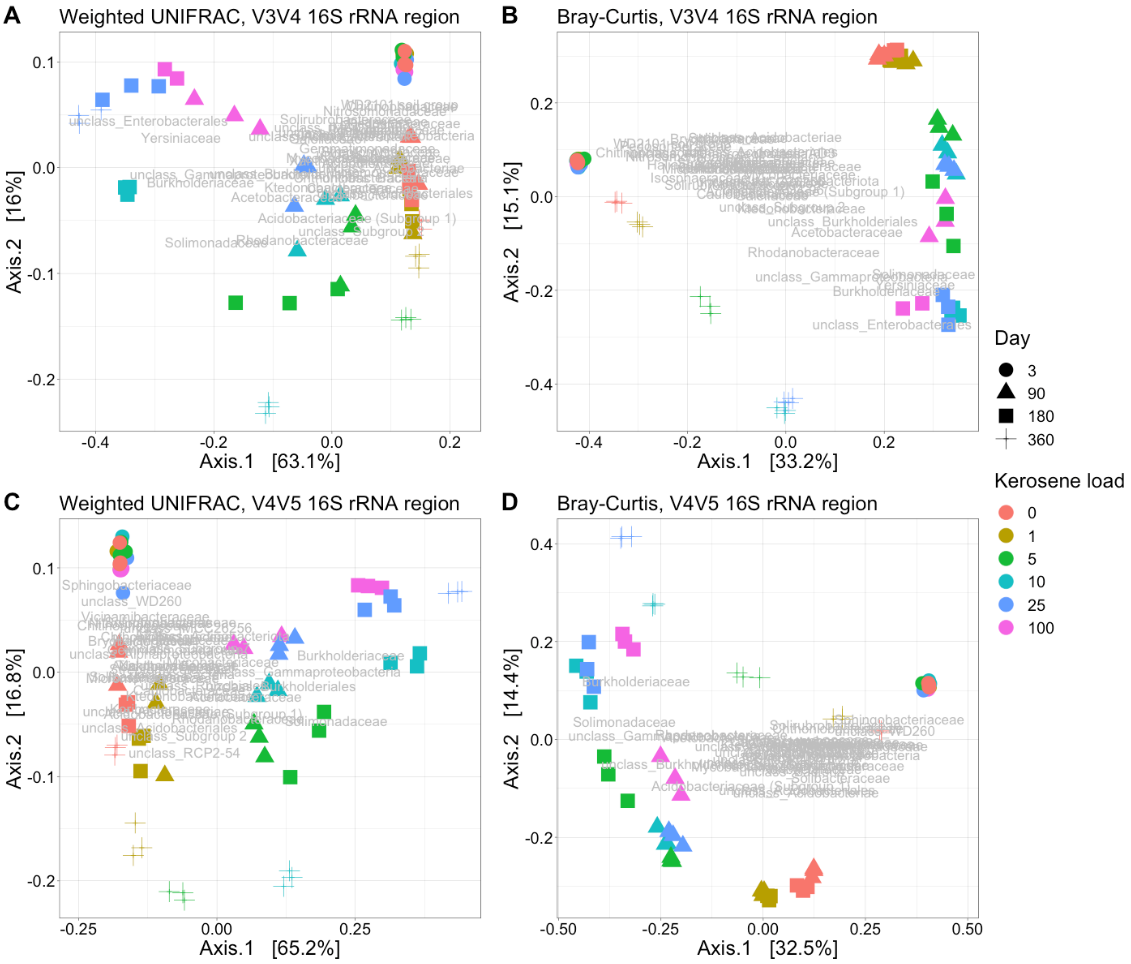

Supplementary Figure S8. Principal coordinate analysis (PCoA) plots for samples of Albic Luvisols (pot experiment).

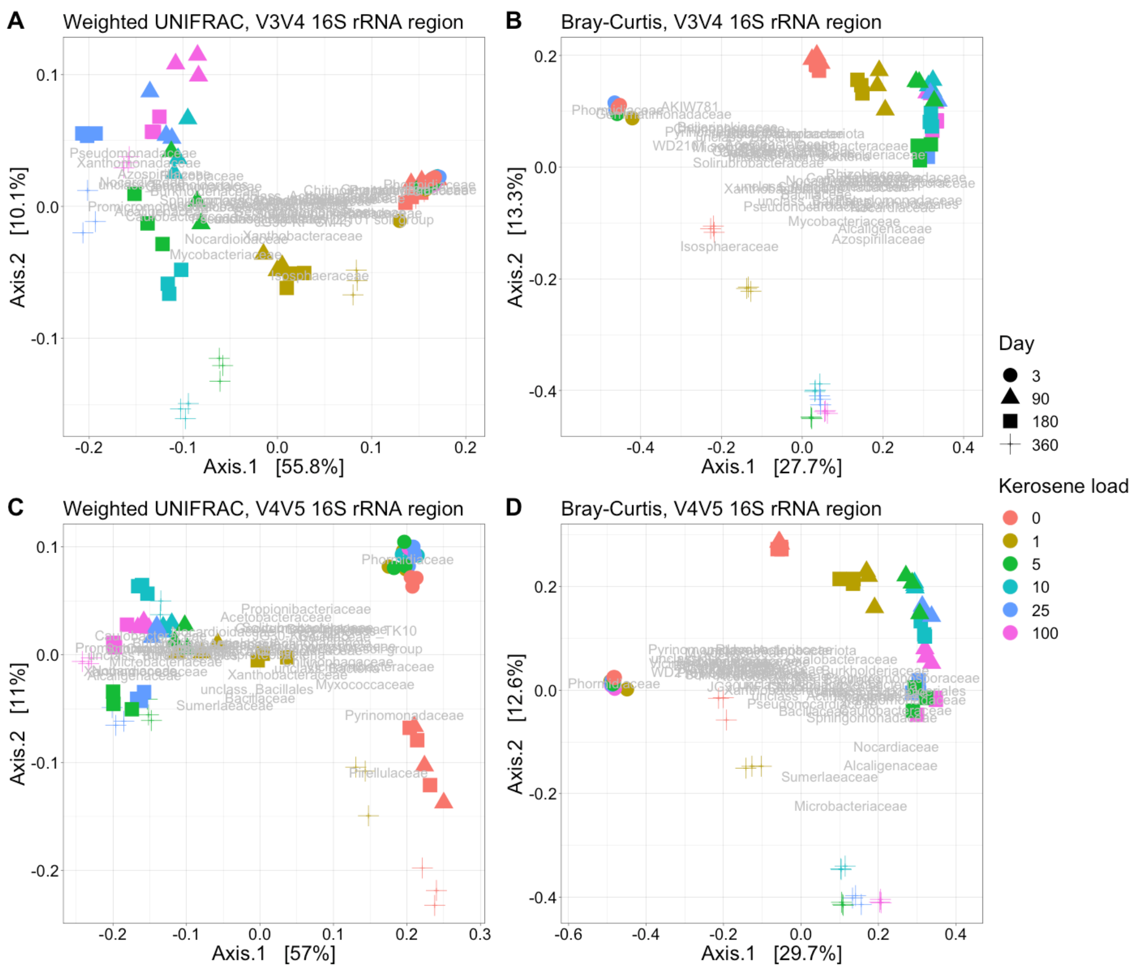

Supplementary Figure S9. Principal coordinate analysis (PCoA) plots for samples of Dystric Arenosols (pot experiment).

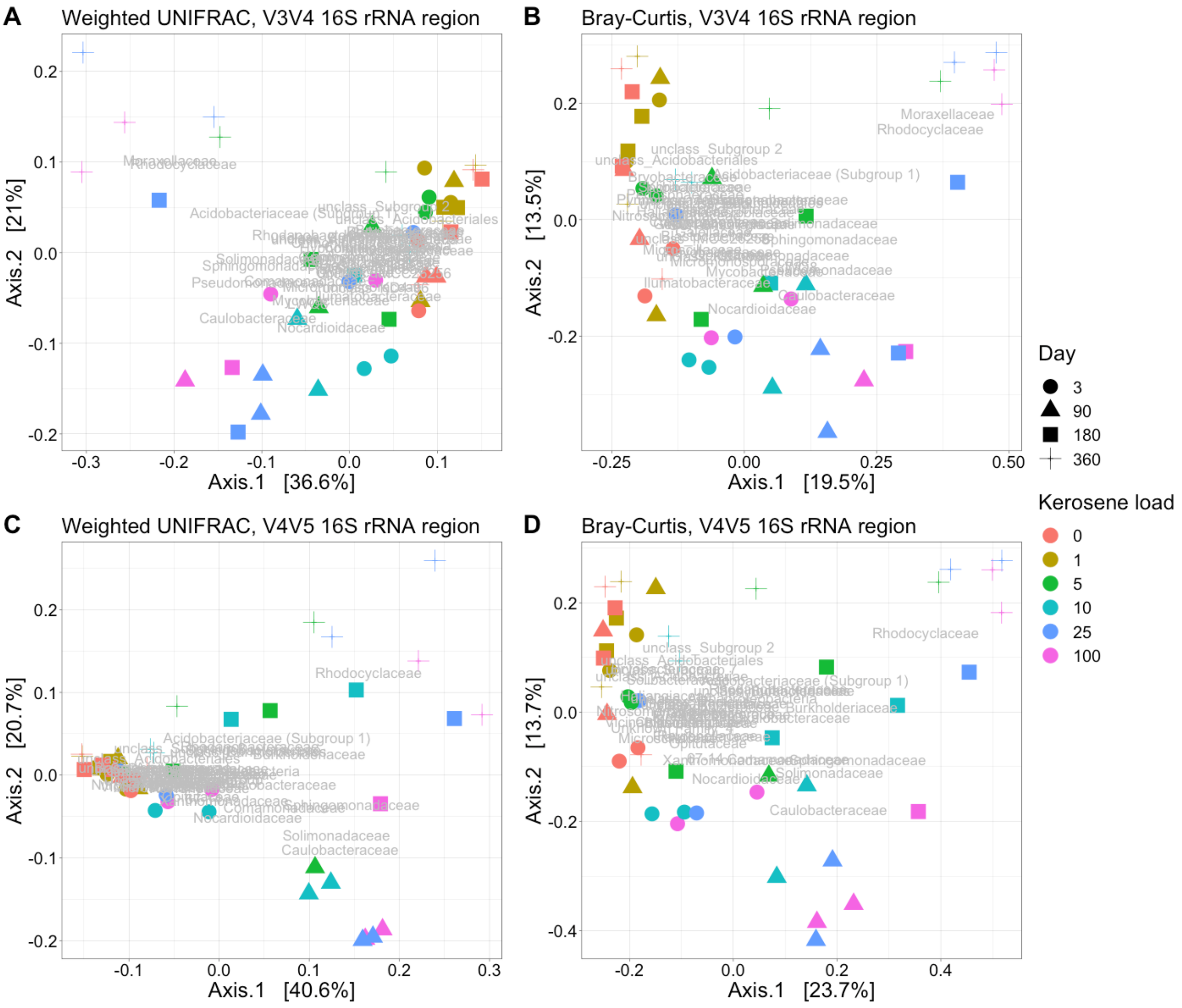

Supplementary Figure S10. Principal coordinate analysis (PCoA) plots for samples of Albic Luvisols (field experiment).

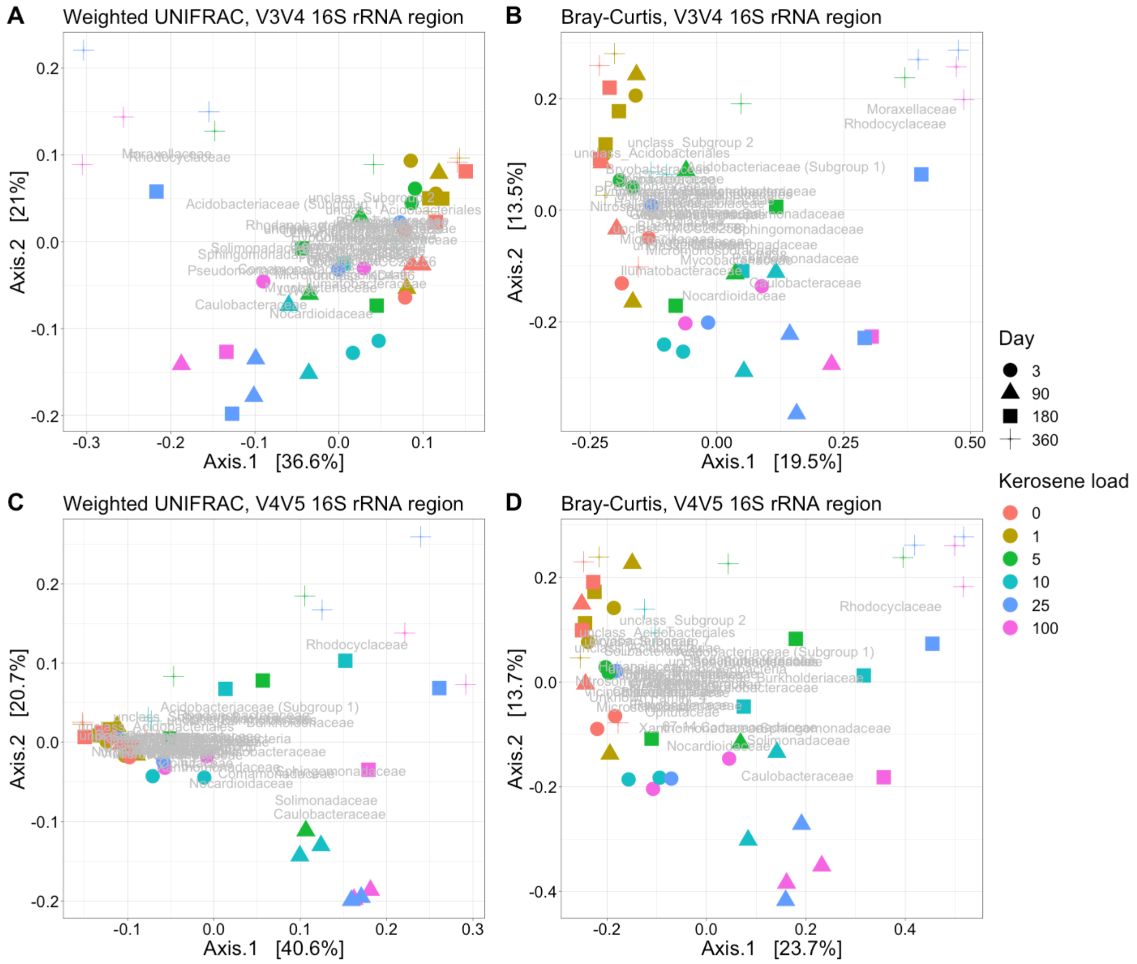

Supplementary Figure S11. Principal coordinate analysis (PCoA) plots for samples of Fibric Histosols (field experiment).

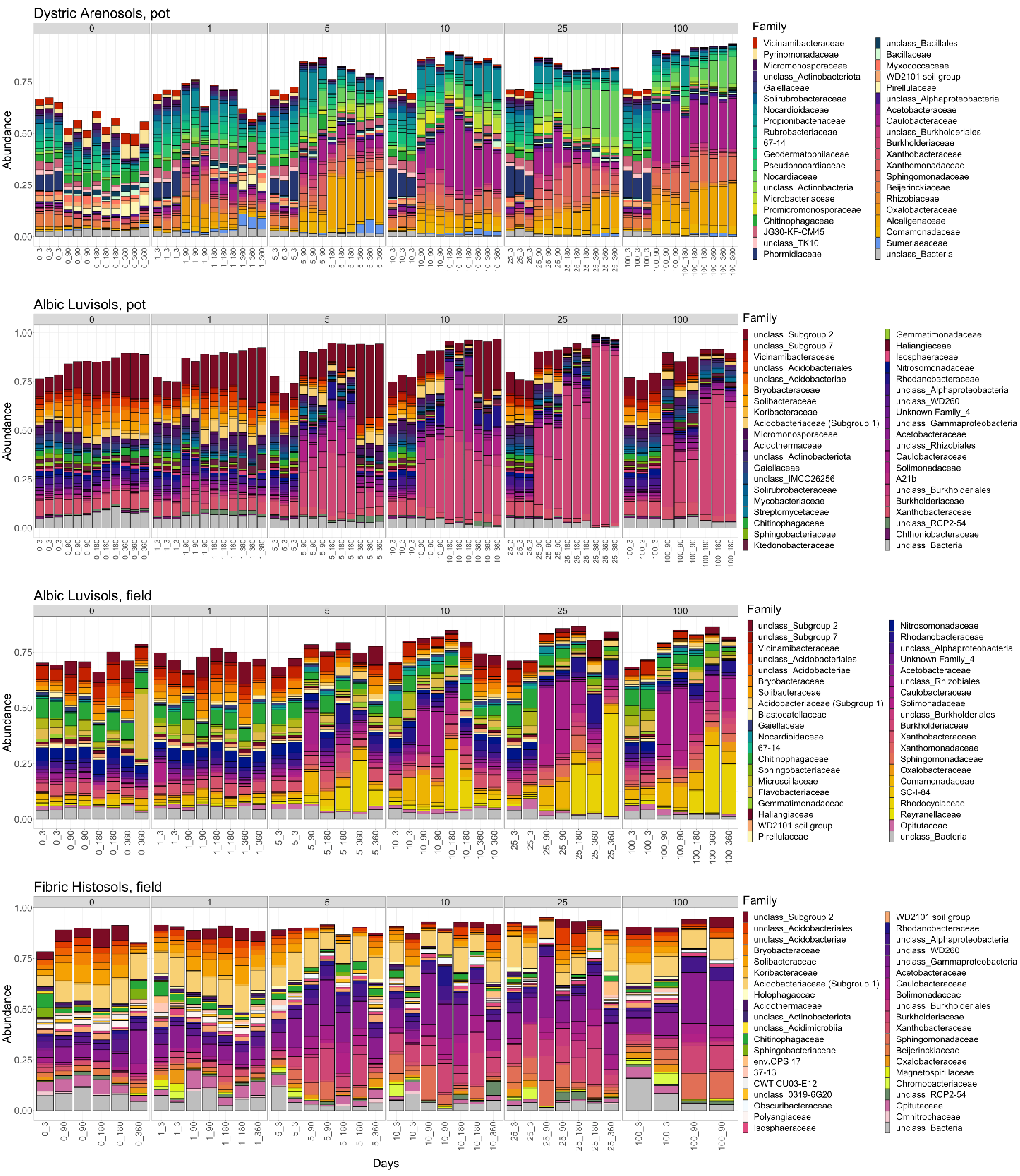

Supplementary Figure S12. Relative abundance of the top forty most frequent bacterial families in the studied soils. The bacterial composition was assessed using the V4V5 region of 16S rRNA.

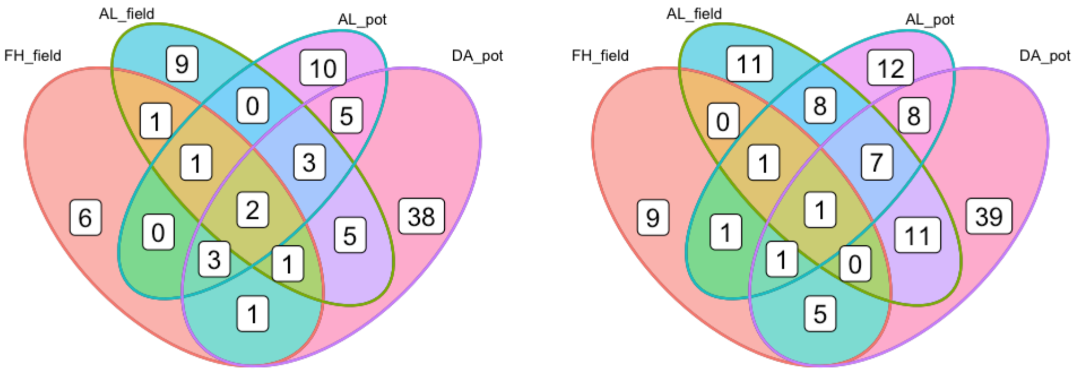

Supplementary Figure S13. The number of common bacterial families significantly increased and decreased in their relative abundance in highly contaminated soil samples.

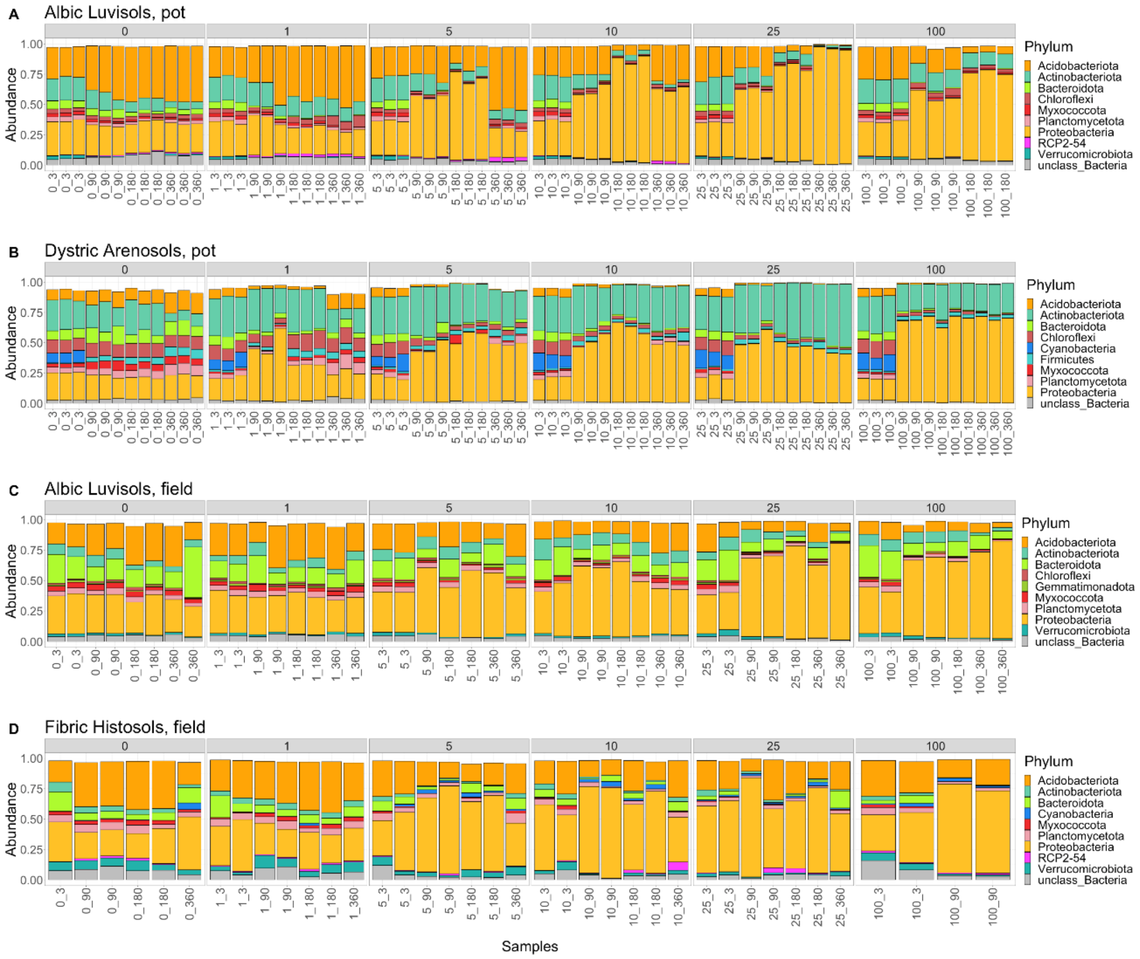

Supplementary Figure S14. Relative abundance of bacterial phyla in the studied soils, assessed using the V4V5 region of 16S rRNA.

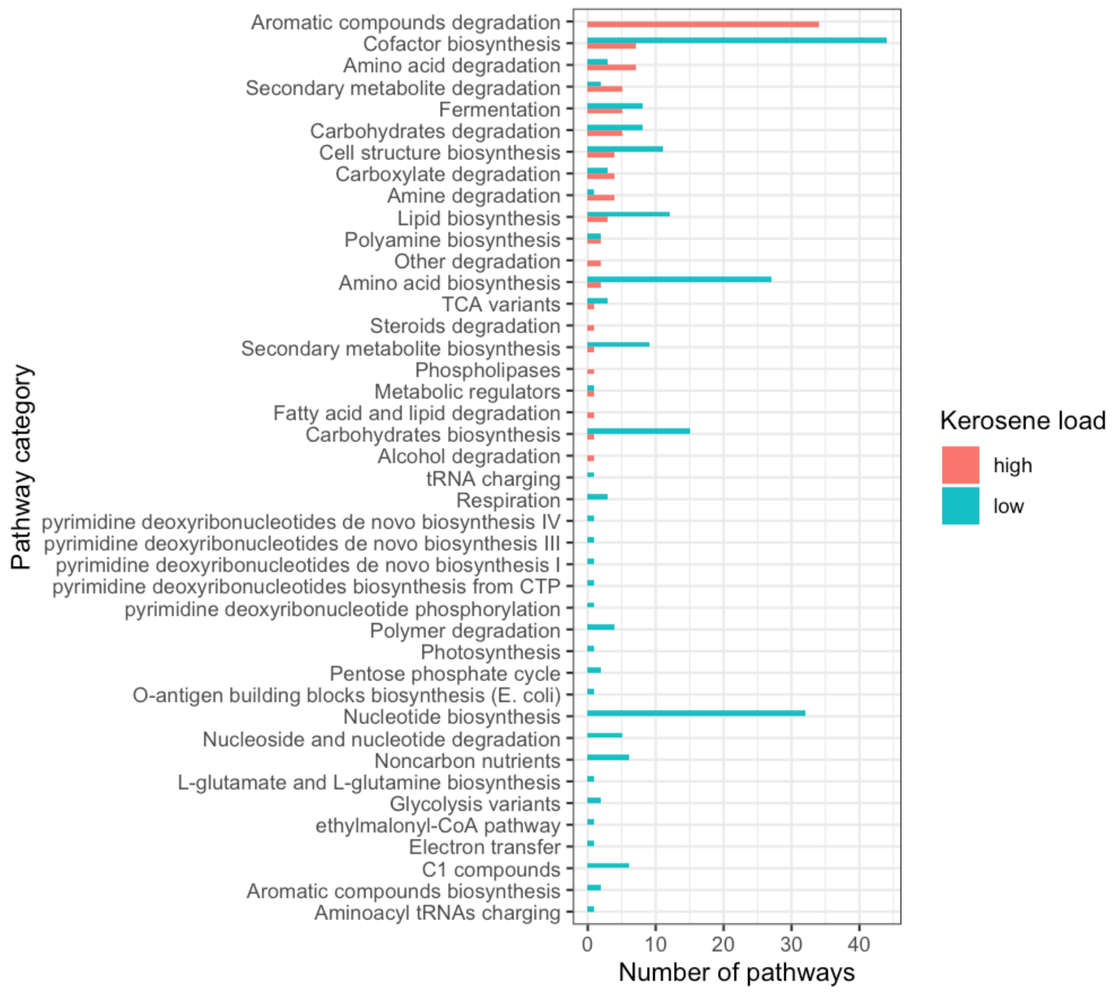

Supplementary Figure S15. Number of differentially abundant MetaCyc pathways from different categories in highly contaminated and slightly contaminated samples in all soils.

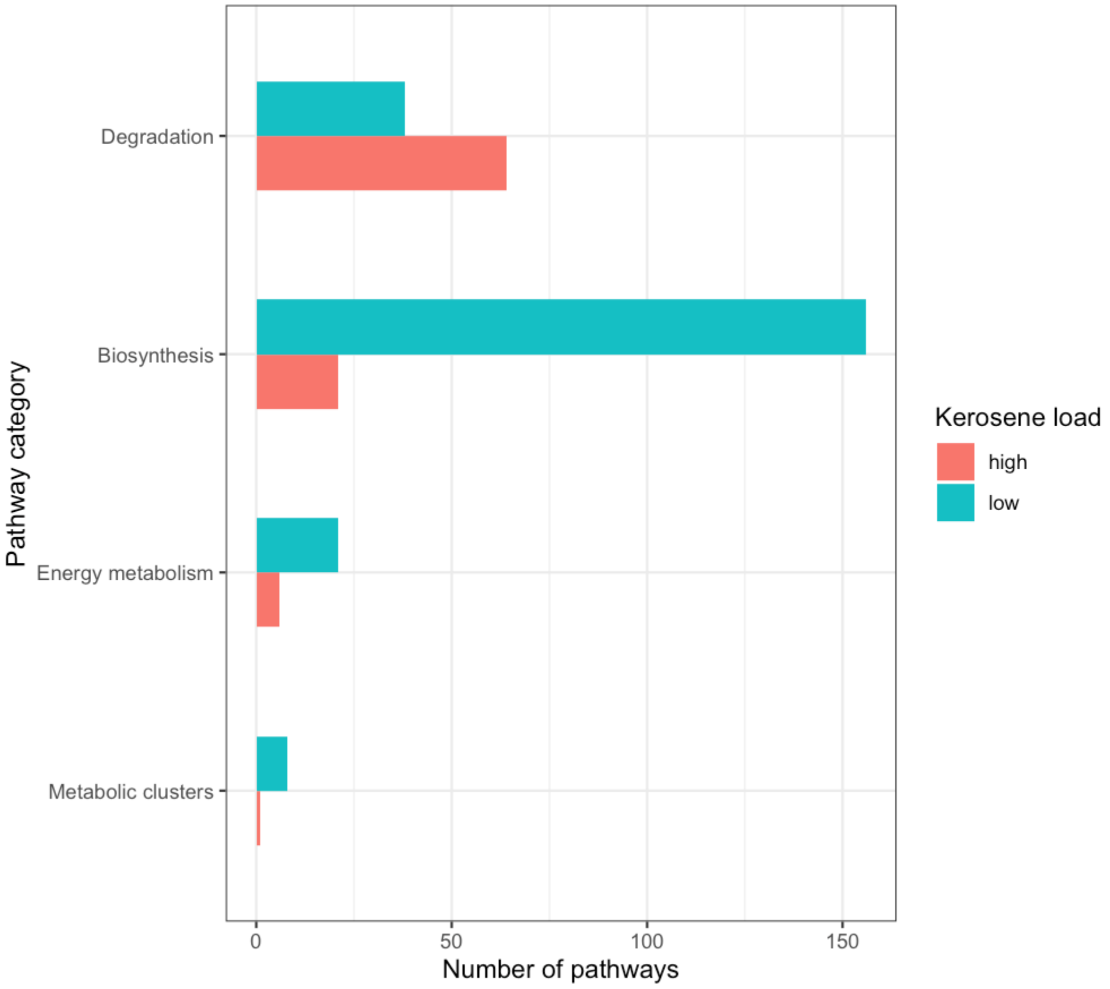

Supplementary Figure S16. Number of differentially abundant MetaCyc pathways from high-level categories in highly contaminated and slightly contaminated samples in all soils.

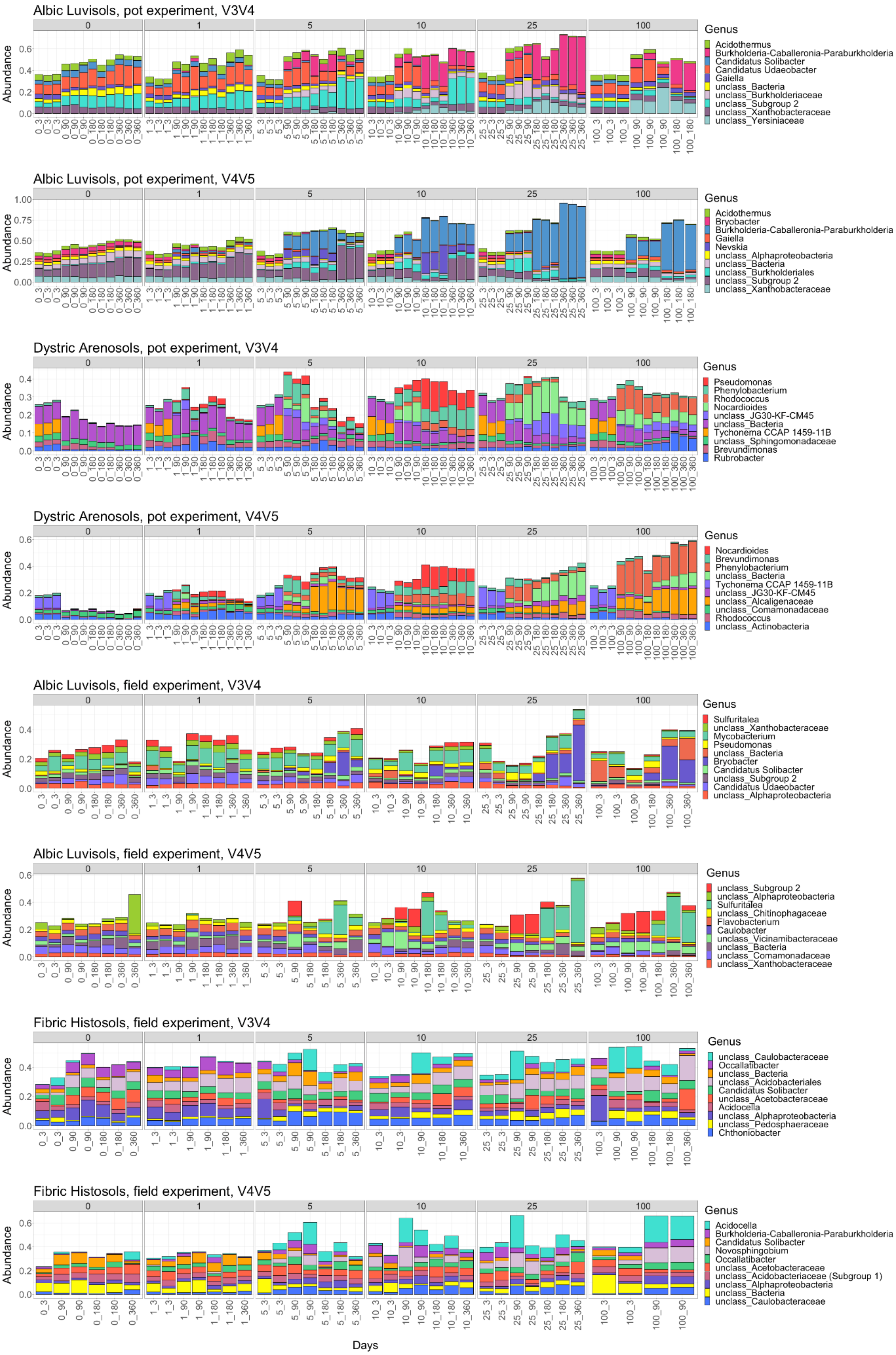

Supplementary Figure S17. Relative abundance of the top ten most frequent bacterial genera in the studied soils. Bacterial composition was assessed using the V3V4 and V4V5 regions of 16S rRNA.

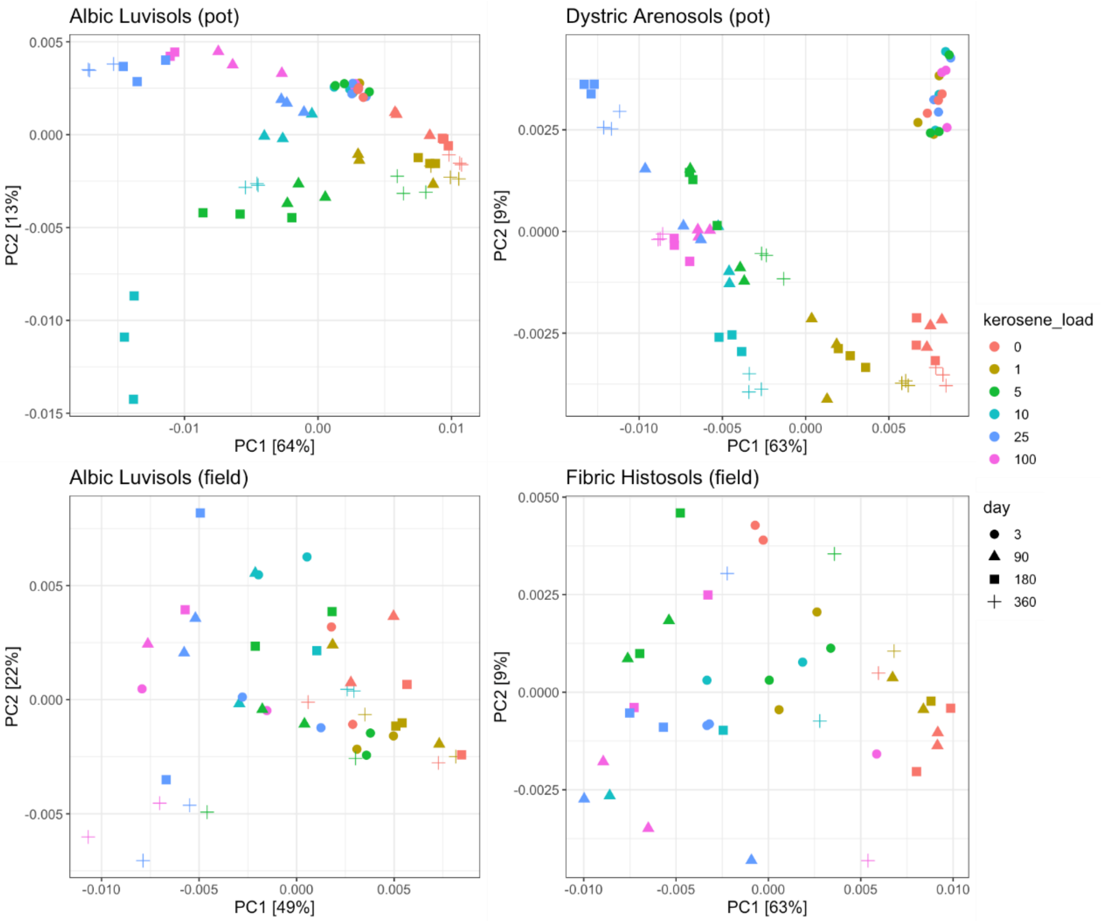

Supplementary Figure S18. PCA plots based on the relative abundance of metabolic pathways predicted with Picrust2 on the V3V4 data.

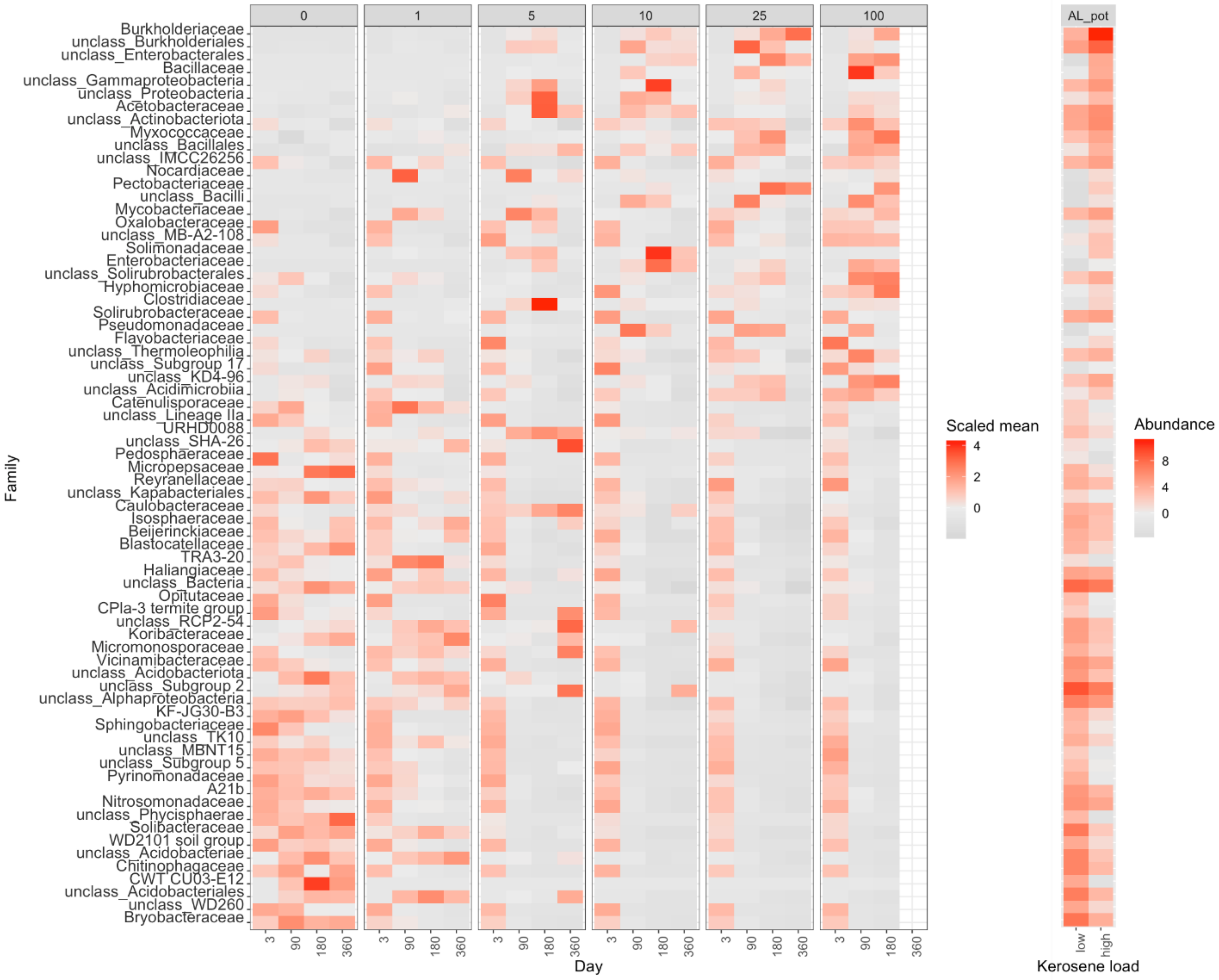

Supplementary Figure S19. Heatmap of the relative abundance of bacterial families that significantly differ in abundance between highly contaminated and control samples of Albic Luvisols, pot experiment. The V4V5 data are shown.

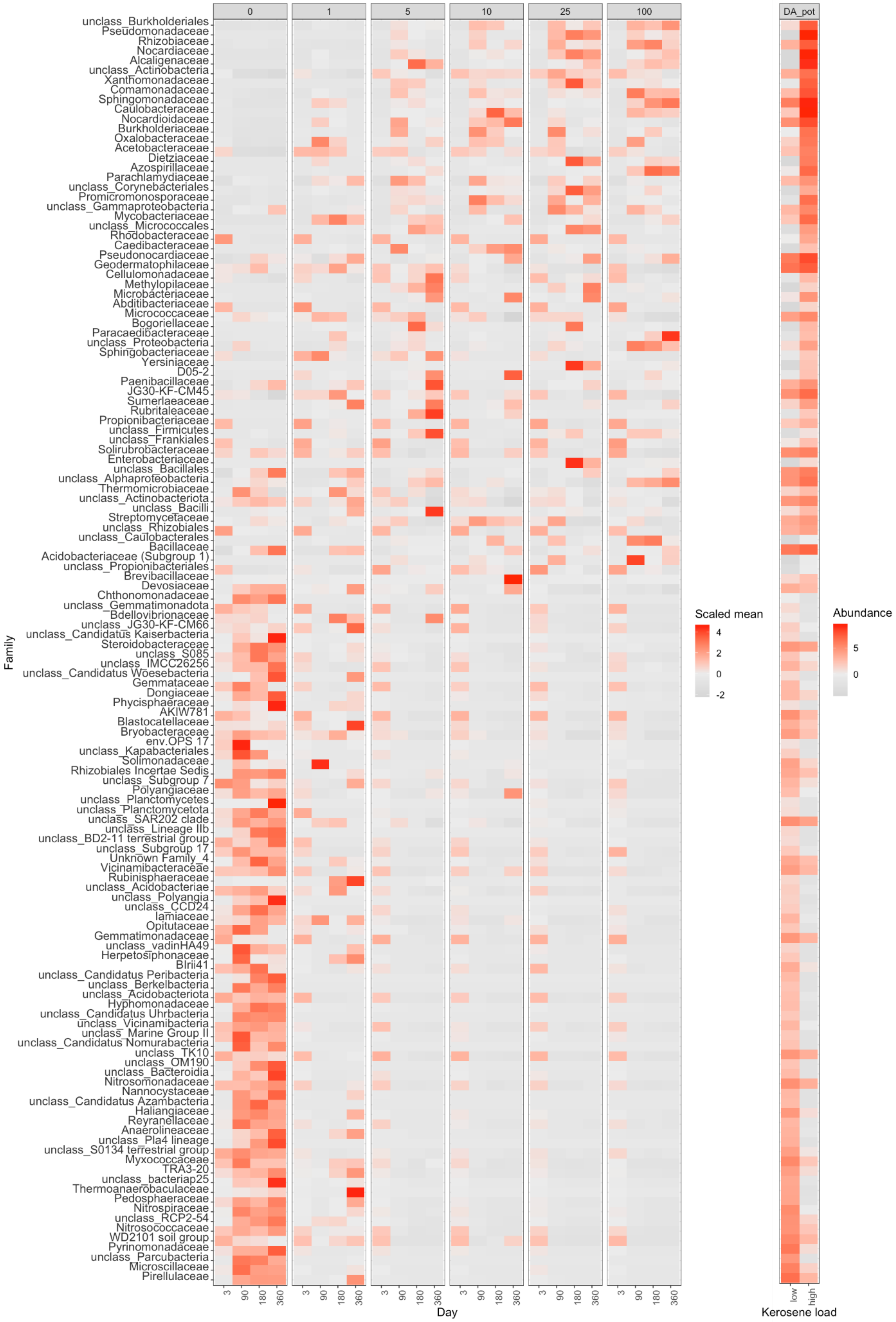

Supplementary Figure S20. Heatmap of the relative abundance of bacterial families that significantly differ in abundance between highly contaminated and control samples of Dystric Arenosol, pot experiment. The V3V4 data are shown.

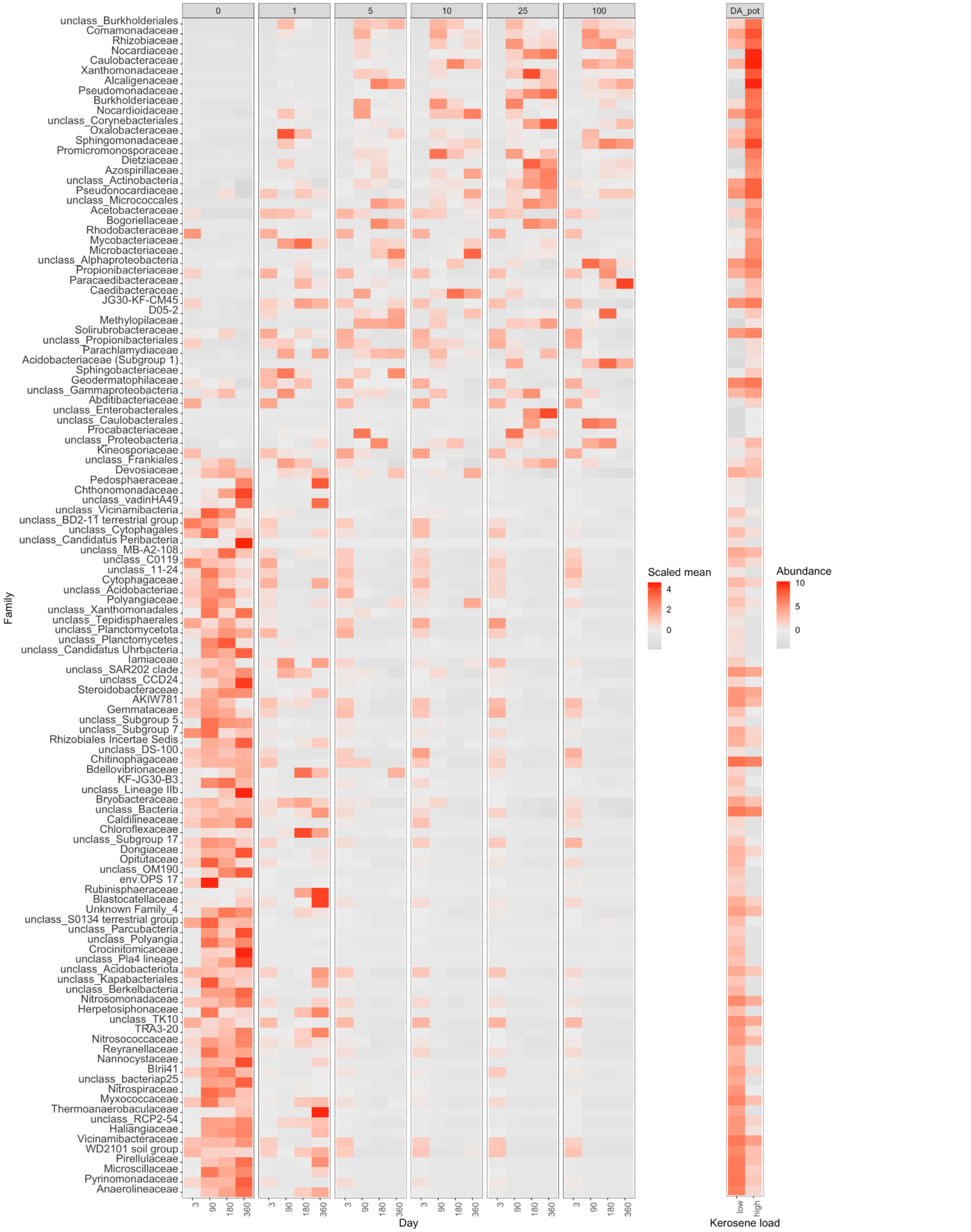

Supplementary Figure S21. Heatmap of the relative abundance of bacterial families that significantly differ in abundance between highly contaminated and control samples of Dystric Arenosol, pot experiment. The V4V5 data are shown.

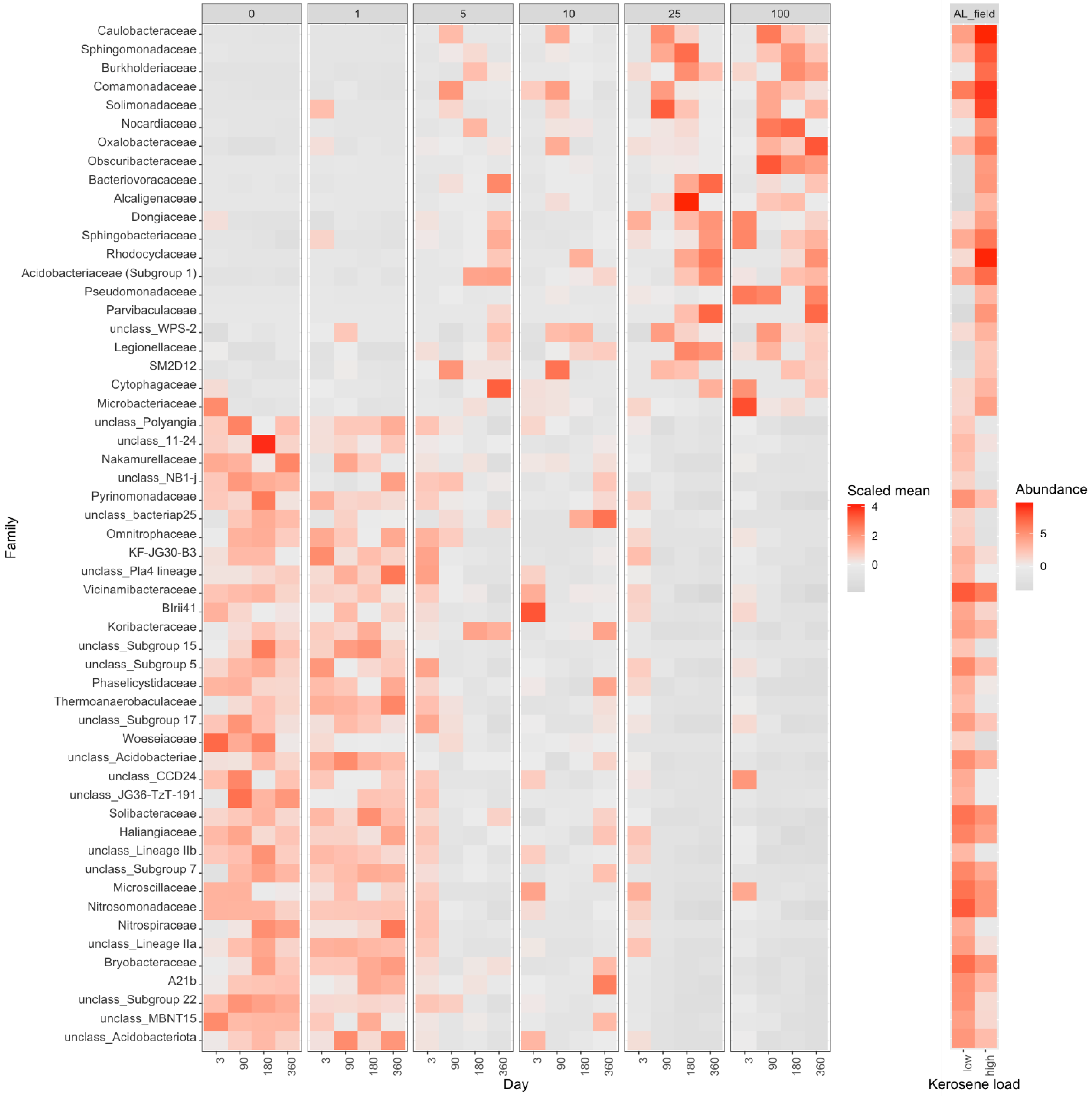

Supplementary Figure S22. Heatmap of the relative abundance of bacterial families that significantly differ in abundance between highly contaminated and control samples of Albic Luvisols, field experiment. The V4V5 data are shown.

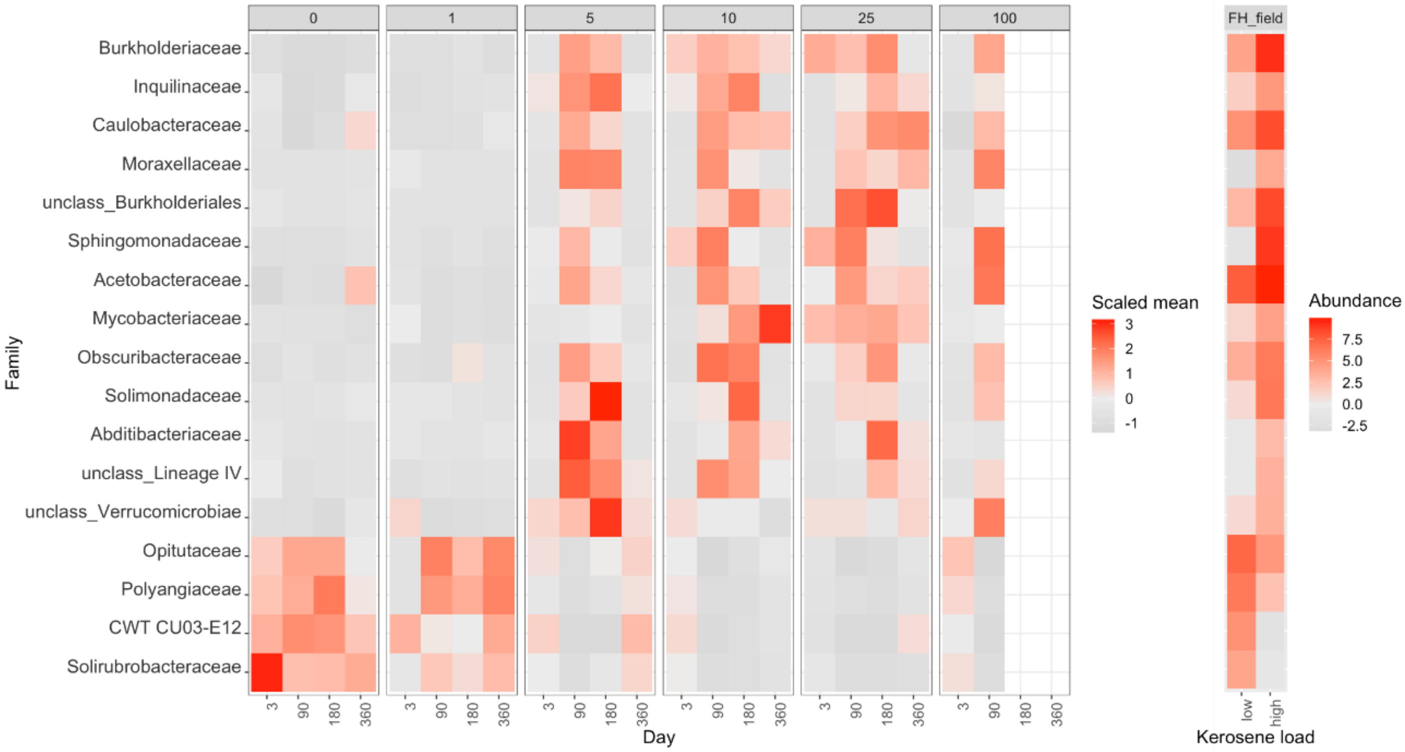

Supplementary Figure S23. Heatmap of the relative abundance of bacterial families that significantly differ in abundance between highly contaminated and control samples of Fibric Histosols, field experiment. The V4V5 data are shown.
